# Effectiveness and Minimal-Invasiveness of Zone 0 Landing Thoracic Endovascular Aortic Repair Using Branched Endograft

**DOI:** 10.3390/jcm11236981

**Published:** 2022-11-26

**Authors:** Tomoaki Kudo, Toru Kuratani, Yoshiki Sawa, Shigeru Miyagawa

**Affiliations:** 1 Department of Cardiovascular Surgery, Osaka University Graduate School of Medicine, Suita 5650871, Osaka, Japan; 2 Department of Minimally Invasive Cardiovascular Medicine, Osaka University Graduate School of Medicine, Suita 5650871, Osaka, Japan

**Keywords:** thoracic endovascular aortic repair, stroke, endoleak

## Abstract

Background: Zone 0 landing thoracic endovascular aortic repair (TEVAR) for the treatment of aortic arch diseases has become a topic of interest. This study aimed to verify whether branced TEVAR (bTEVAR) is an effective and a more minimally invasive treatment by comparing the outcomes of bTEVAR and hybrid TEVAR (hTEVAR) in landing zone 0. Methods: This retrospective, single-center, observational cohort study included 54 patients (bTEVAR, *n* = 25; hTEVAR, *n* = 29; median age, 78 years; median follow-up period, 5.4 years) from October 2012 to June 2018. The logistic Euro-SCORE was significantly higher in the bTEVAR group than in the hTEVAR group (38% vs. 21%, *p* < 0.001). Results: There was no significant difference the in-hospital mortality between the bTEVAR and hTEVAR groups (0% vs. 3.4%, *p* = 1.00). The operative time (220 vs. 279 min, *p* < 0.001) and length of hospital stay (12 vs. 17 days, *p* = 0.013) were significantly shorter in the bTEVAR group than in the hTEVAR group. The 7-year free rates of aorta-related deaths (bTEVAR [95.5%] vs. hTEVAR [86.9%], *Log-rank* *p* = 0.390) and aortic reintervention (bTEVAR [86.3%] vs. hTEVAR [86.9%], *Log-rank* *p* = 0.638) were not significantly different. Conclusions: The early and mid-term outcomes in both groups were satisfactory. bTEVAR might be superior to hTEVAR in that it is less invasive. Therefore, bTEVAR may be considered an effective and a more minimally invasive treatment for high-risk patients.

## 1. Introduction

Conventional open surgery is the most commonly performed procedure for aortic arch diseases. However, the treatment of aortic arch pathologies is complicated to treat because conventional open surgeries are highly invasive and complex [1,2]. In some reports, the short-term results of hybrid thoracic endovascular aortic repair (TEVAR) were superior to those of conventional open surgery, whereas the long-term outcomes were equivalent [3,4,5]. Recently, hybrid TEVAR (hTEVAR) has gained increasing attention for the treatment of aortic arch pathologies, especially for high-risk patients [6,7,8]. Conventional zone 0 landing hTEVAR is moderately invasive surgical procedure because it requires median sternotomy and aorto-cervical bypasses but does not necessitate cardiopulmonary bypass [9,10,11]. To reduce the invasiveness, we have performed TEVAR using a branched stent-graft, in which complex aorto-cervical bypass or graft replacement is not required [12]. The purpose of this study was to verify whether branched (bTEVAR) is an effective and a more minimally invasive treatment by comparing the outcomes of bTEVAR and hTEVAR in the landing zone (LZ) 0.

## 2. Materials and Methods

### 2.1. Ethics Statement and Study Design

All protocols of TEVAR using branched endografts in this study were approved by the Medical Ethics Committee of Osaka University School of Medicine (No. 15087). Informed consent was obtained from all patients before the procedures. This study was conducted as a single-center, retrospective, and observational cohort study.

### 2.2. Preoperative Measurements and Treatment Strategy

All patients underwent contrast-enhanced multidetector computed tomography (MDCT) using a slice thickness of ≤1 mm. Three-dimensional reconstructions were performed on an image processing workstation (Aquarius Intuition; TeraRecon, Foster City, CA, USA) to evaluate the adequacy of the proximal and distal LZs, the inflow artery, the aortic arch, and the access vessels before the procedure. The indications for surgical intervention were aortic diameter expansion by ≥5 mm in six months, a maximum aortic diameter of >55 mm, aortic rupture, any size of saccular aneurysm, a malperfusion syndrome, or an initial diameter of >40 mm in type B aortic dissection. We did not perform a hTEVAR with a zone 0 landing in patients with suitable LZs at zones 1 and 2. In the treatment strategy, we ensured the following preprocedural conditions: proximal LZ diameter was ≤42 mm, and atheroma grade of the proximal LZ and the cervical arteries was 1 or 2, as described previously [6,7].

### 2.3. Surgical Procedure

#### 2.3.1. Branched TEVAR

First, the patients received an extra-anatomical bypass from the right axillary artery (AxA) to the left AxA or from the right AxA to the left common carotid artery (CCA) and the left AxA using a ringed 8 mm expanded polytetrafluoroethylene graft. To protect against embolization, the left subclavian artery (LSA) was occluded using the balloon catheter.

A curved super-stiff wire was advanced to the left ventricle. The custom-made Bolton Relay NBS stent-graft (Bolton Medical, Inc., Sunrise, FL, USA) device was inserted through the femoral artery. We confirmed the precise match between the orifices of the cervical arteries and the device gate by performing standard angiography and 3D mapping using the Dyna-CT. Rapid pacing (heart rate > 160 bpm) was started, and the main device was deployed at a constant speed. Next, the wire was advanced to the posterior tunnel from the right CCA, and the stent-graft for the brachiocephalic artery (BCA) was inserted into the tunnel and deployed. The stent-graft deployment in the left CCA was performed by the same procedure. Lastly, we performed coiling of the left subclavian artery. Aortography was conducted to check for endoleaks and bypass patency, as described previously, as described previously [12].

#### 2.3.2. Hybrid TEVAR

After median sternotomy, end-to-side anastomosis was performed using a woven Dacron trifurcated graft on the greater curvature of the ascending aorta with a partial occlusion clamp without cardiopulmonary bypass. Subsequently, the BCA and left CCA were anastomosed to the branch of the graft in an end-to-end manner. The LSA was anastomosed side-to-end. The BCA was occluded by suturing, the left CCA was clipped, and the LSA was clipped or embolized with a coil. In the case of banding the ascending aorta, a woven Dacron graft was cut to the target length, and the ascending aorta was shrunk. After the supra-aortic vessels were rerouted, stent-graft devices were deployed, as described previously [7].

#### 2.3.3. Follow-Up

Follow-up was performed at our department during regular patient visits at least once every 3 months for the first year and every 6 months or annually thereafter. MDCT was performed before discharge, at 6 months after the procedure, and yearly thereafter. Patients were followed up until death, the details of which were confirmed through telephonic interviews with their families.

Aortic events included known or suspected events such as aortic diameter enlargement >5 mm, any endoleaks, stent-graft migration, aneurysm rupture, aortic dissection, bypass graft occlusion, and prosthetic infection. Aorta-related deaths were defined as death due to aortic reinterventions.

### 2.4. Statistical Analyses

Results are expressed as mean ± standard deviation and median (interquartile range [IQR]) according to the normality of distribution as assessed using the Shapiro–Wilk test and were compared using the Mann–Whitney U test. Categorical variables, presented as counts and percentages, were analyzed using the chi-square test or the Fisher’s exact test. The curves for overall survival and freedom from aorta-related death, aortic events, and aortic reintervention were estimated using the Kaplan–Meier product-limiting method and compared using the *Log-rank* test. Estimates were provided with 95% confidence intervals (CIs). All *p*-values were two-sided, and *p* < 0.05 was considered to indicate statistical significance. All statistical analyses were performed using JMP statistical software, version 16.0.0 for MacOS X (SAS Institute Inc., Cary, NC, USA).

## 3. Results

### 3.1. Study Population

**Figure 1:**
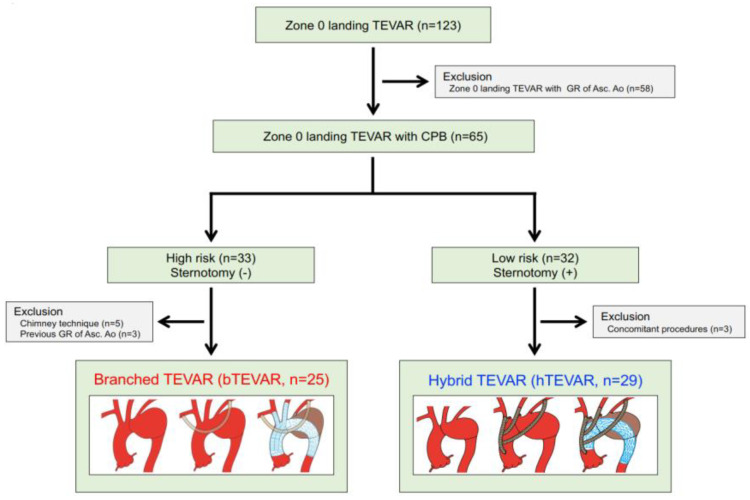
Treatment algorithm of this study. TEVAR: thoracic endovascular aortic repair; GR: graft replacement; Asc. Ao: ascending aorta; bTEVAR: branched TEVAR; hTEVAR: hybrid TEVAR.

The patient flow diagram is shown in Figure 1. Of 123 patients who underwent zone 0 landing TEVAR from October 2012 to June 2018, 54 underwent zone 0 landing TEAVR were included in this study: bTEVAR (*n* = 25, 46.3%) in patients with incapable of median sternotomy and hTEVAR (*n* = 29, 53.7%) in patients with capable of median sternotomy. We excluded cases with zone 0 landing TEVAR with graft replacement of the ascending aorta, chimney technique, graft replacement of the ascending aorta for aneurysm and type A dissection, and concomitant procedures. No patients were lost to follow-up, and all patient data were available.

### 3.2. Patients’ Characteristics

The patient characteristics are listed in Table 1. The median follow-up period was 5.4 years (IQR, 3.2–7.8 years). The median patient age at surgery was 78 years (IQR, 73–82 years), 22 (40.7%) patients were older than 80 years, and 12 (22.2%) patients were female. None of the patients underwent emergent procedures. The pathologies were attributed to dissecting aortic aneurysms in five (9.3%) patients; however, no patients had a patent false lumen. Thirteen (24.1%) patients had a history of cardiovascular surgery, however, no patients had previous median sternotomy. The median logistic Euro-SCORE was 32% (IQR, 20–40%). The logistic Euro-SCORE was significantly higher in the bTEVAR group (38%; IQR, 34–56%) than in the hTEVAR group (21%; IQR, 13–30%) (*p* < 0.001).

### 3.3. Preoperative Measurements and Stent-Grafts

The preoperative measurements obtained by contrast-enhanced MDCT are shown in Table 2. The median maximum aneurysmal diameter was 58 mm (IQR, 53–65 mm). The median length and diameter of the proximal LZ were 33.6 ± 6.8 mm and 33.6 ± 3.0 mm, respectively. The mean length of the proximal LZ was significantly longer in the bTEVAR group (35.6 ± 1.3 mm) than in the hTEVAR group (31.9 ± 5.4 mm) (*p* = 0.049). The mean diameter of the proximal LZ was significantly greater in the bTEVAR group (39.4 ± 3.5 mm) than in the hTEVAR group (32.5 ± 2.0 mm) (*p* = 0.003).

The numbers of patients with an atheroma grade of ≥2 in the ascending aorta and BCA were 13 (24.1%) and 14 (25.9%), respectively. These numbers were not significantly different between the two groups.

For proximal stent grafting, the custom-made branched Relay NBS was used in 25 (46.3%) patients in the bTEVAR group. The Bolton Relay Plus (Bolton Medical, Inc.) was used in two (3.7%) patients, Gore TAG (W.L. Gore & Associates, Inc., Flagstaff, AZ, USA) in 10 (18.5%) patients, Gore CTAG (W.L. Gore & Associates, Inc.) in 16 (29.6%) patients, and Cook Zenith TX2 (Cook Medical, Inc., Bloomington, IN, USA) in one (1.9%) patient in the hTEVAR group. The mean size of the proximal stent-graft was 39.2 ± 3.8 mm, and the mean oversizing rate was 16.9 ± 8.1%. The mean size of the proximal stent-graft was significantly larger in the bTEVAR group (41.9 ± 3.3 mm) than of the hTEVAR group (36.9 ± 2.4 mm) (*p* < 0.001), and the mean oversizing rate of the proximal stent-graft was significantly larger in the bTEVAR group (20.7 ± 8.1 %) than of the hTEVAR group (13.7 ± 6.6%) (*p* < 0.001).

### 3.4. Operative and in-Hospital Outcomes

The operative and in-hospital data are shown in Table 3. All procedures were successful, and the median operative time was 255 min (IQR, 217–290 min). The operative time was significantly longer in the hTEAVR group (220 min; IQR, 193–257 min) than in the bTEVAR group (279 min; IQR, 246–328 min) (*p <* 0.001). The median postoperative hospital stay was 16 days (IQR, 12–25 days). The postoperative hospital stay was significantly longer in the hTEVAR group (17 days; IQR, 14–26 days) than in the bTEVAR group (12 days; IQR, 9–22 days) (*p* = 0.013).

One (1.9%) patient in the hTEVAR group had 30-day mortality due to retrograde type A dissection (RTAD). Two (3.7%) patients experienced permanent neurological dysfunction (PND). There were no patients with endoleaks. One (1.9%) patient in the hTEVAR group had in-hospital aortic event due to retrograde type A dissection (RTAD). However, the patient died at 11 days after hTEVAR.

### 3.5. Late Outcomes

The late outcomes are presented in Table 4. Aneurysm rupture was detected in two (3.7%) patients in the bTEVAR group. Those two patients had stent-graft migration, which was the cause of type 1b and 3 endoleaks. Of these two patients, the one patient with type 3 endoleak survived, whereas the other did not. Type 1a endoleak and RTAD were not observed in the late stage. Four (7.4%) cases had aortic events due to two (3.7%) aneurysm ruptures and two (3.7%) prosthetic infections.

### 3.6. Survival

**Figure 2:**
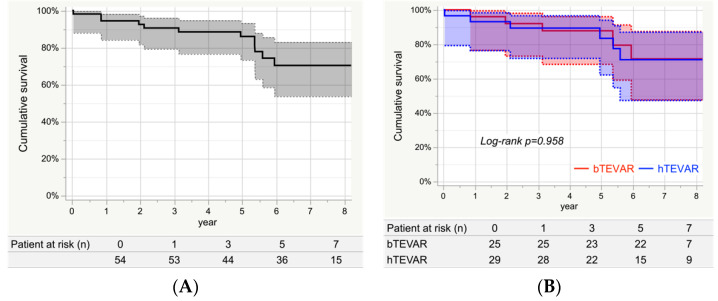
Cumulative survival. (**A**) Cumulative survival of the entire study group. The cumulative survival rates at 1, 3, 5, and 7 years were 94.4% (95% CI:84.1–98.2%), 90.6% (79.3–96.0%), 86.0% (73.3–93.2%), and 70.4% (53.7–83.0%), respectively. (**B**) Cumulative survival of each group. The 7-year survival rates in the bTEVAR group and the hTEVAR group were 71.5% (47.6–87.4%) and 71.0% (47.3–87.0%), respectively. There were no significant differences between the two groups (*Log-rank* *p* = 0.958).

The Kaplan–Meier curve indicating the cumulative survival is presented in Figure 2A. The survival rates at 1, 3, 5, and 7 years were 94.4% (95% CI: 84.1–98.2%), 90.6% (95% CI: 79.3–96.0%), 86.0% (95% CI: 73.3–93.2%), and 70.4% (95% CI: 53.7–83.0%), respectively. Figure 2B shows that the survival rates at 7 years were 71.5% (95% CI: 47.6–87.4%) and 71.0% (95% CI: 47.3–87.0%) for bTEVAR and hTEVAR groups, respectively, which were not significantly different (*Log-rank* *p* = 0.958).

### 3.7. Aorta-Related Death

**Figure 3:**
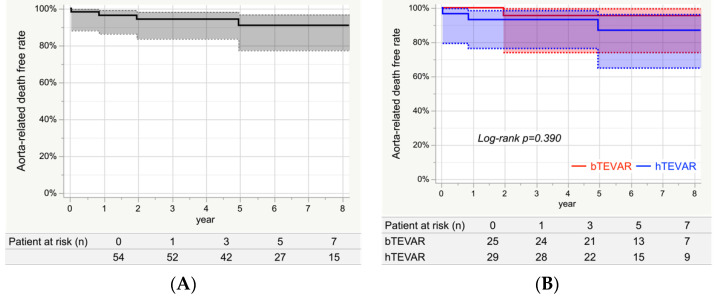
Freedom from aorta-related death. (**A**) Freedom from aorta-related deaths in the entire study group. The event-free rates at 1, 3, 5, and 7 years were 96.3% (95% CI: 86.2–99.1%), 94.2% (95% CI: 83.5–98.1%), 90.8% (95% CI: 77.2–96.7%), and 90.8% (95% CI: 77.2–96.7%), respectively. (**B**) Freedom from aorta-related deaths in each group. The aorta-related death free rates at 7 years for the bTEVAR and hTEVAR groups were 95.5% (95% CI: 73.9–99.4%) and 86.9% (95% CI: 64.8–96.0%), respectively, which were not significantly different (*Log-rank* *p* = 0.390).

Figure 3A shows the Kaplan–Meier curve indicating aorta-related death for the entire study group. The event-free rates at 1, 3, 5, and 7 years were 96.3% (95% CI: 86.2–99.1%), 94.2% (95% CI: 83.5–98.1%), 90.8% (95% CI: 77.2–96.7%), and 90.8% (95% CI: 77.2–96.7%), respectively. During the follow-up period, there were four aorta-related deaths, including one patient in the bTEVAR group who developed aneurysm rupture due to a type 1b endoleak, and in the hTEVAR group, one patient who had RTAD and two patients had the prosthetic infection. Figure 3B shows that the aorta-related death-free rates at 7 years for the bTEVAR and hTEVAR groups were 95.5% (95% CI: 73.9–99.4%) and 86.9% (95% CI: 64.8–96.0%), respectively, which were not significantly different (*Log-rankp* = 0.390).

### 3.8. Aortic Events

**Figure 4:**
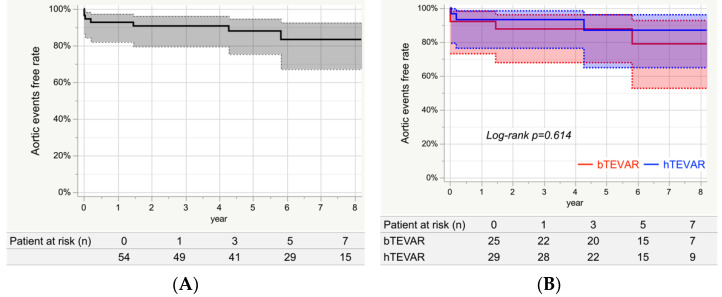
Freedom from aortic events. (**A**) The event-free rates at 1, 3, 5, and 7 years were 92.6% (95% CI: 81.9–97.2%), 90.6% (95% CI: 79.4–96.0%), 87.9% (95% CI: 75.2–94.5%), and 83.3% (95% CI: 67.1–92.4%), respectively. (**B**) The aortic events-free rates at 7 years for the bTEVAR and hTEVAR groups were 78.9% (95% CI: 52.7–92.6%) and 86.9% (95% CI: 64.8–96.0%), respectively, with no significant differences (*Log-rank* *p* = 0.614).

Figure 4A shows the Kaplan–Meier curve indicating aortic events for the entire study group. The event-free rates at 1, 3, 5, and 7 years were 92.6% (95% CI: 81.9–97.2%), 90.6% (95% CI: 79.4–96.0%), 87.9% (95% CI: 75.2–94.5%), and 83.3% (95% CI: 67.1–92.4%), respectively. Seven patients had aortic events: two had PND and one had RTAD in the early phase, and two had aneurysm rupture due to type 1b and 3 endoleaks and two had prosthetic infection in the late phase. Figure 4B shows the Kaplan–Meier event-free curves stratified by group. The aortic event-free rates at 7 years for the bTEVAR and hTEVAR groups were 78.9% (95% CI: 52.7–92.6%) and 86.9% (95% CI: 64.8–96.0%), respectively, with no significant differences (*Log-rankp* = 0.614).

## 4. Discussion

For over a half of a century, the conventional total arch replacement has been considered the gold standard for the surgical treatment of aortic arch diseases [13,14,15]. However, this treatment is difficult for high-risk patients due to its complexity and substantial invasiveness, thus yielding unsatisfactory outcomes such as an in-hospital mortality rate of 5.0–11.3%, as reported by some studies [9,16,17,18]. TEVAR was introduced as a minimally invasive technique, while hTEVAR was reported as a potential alternative to conventional total arch replacement in high-risk patients [3,6,7,8,19,20,21]. Milewski et al. [22] stated that the high-risk patients aged >75 years had significantly lower in-hospital mortality after hybrid TEVAR. Consequently, the indications for hybrid TEVAR have been gradually expanded. The previously reported the in-hospital mortality after zone 0 landing TEVAR was 5.0–12% [9,23,24,25]. In fact, some reports from institutions performing both conventional arch repair and hTEVAR stated that the in-hospital mortalities were not significantly different [9,19,24,26]. In this study, early results were satisfactory because the in-hospital mortality was 1.6% (*n* = 1).

Because bTEVAR had a shorter operation time and postoperative hospital stay (bTEVAR: 12 days vs. hTEVAR: 17 days; *p* = 0.013), bTEVAR is considered to be less invasive than hTEVAR. In addition, bTEVAR could be cosidered an effective and a more minimally invasive treatment despite its use in high-risk patients, because the early and mid-term results of bTEVAR and hTEVAR are not significantly different. The PND rates in the bTEVAR and hTEVAR groups were 8.0% (*n* = 2) and 0%, respectively. The PND rate in the bTEVAR group reported by previous articles was equal to that of zone 0 landing hTEVAR (5–17%); however, it was higher than that reported in previous studies with conventional arch repair (2–9%) [10,18,19,20,21,22,23,24]. In addition, the 7-year aorta-related death-free and aortic event-free rates were 90.8% (bTEVAR: 95.5% and hTEVAR: 86.9%, *Log-rank* *p* = 0.390) and 83.3% (bTEVAR: 78.9% and hTEVAR: 86.9%, *Log-rank* *p* = 0.614), which were equal to those of conventional arch repair [13,14]. In the future, it will be possible to reduce the invasiveness of the surgery by performing bTEVAR; however, PND and aortic reintervention due to endoleak should be prevented.

As for PND, preoperative evaluation of aorta properties, such as shagginess, is important, as reported in other studies [27]. We reported that the risk factor for PND in bTEVAR is an atheroma grade ≥2 in the BCA, and we recommend hTEVAR instead of bTEVAR for such patients [12].

Type 1a endoleaks are a fatal complication to be avoided, with few practical alternatives to endovascular treatment. In previous reports, the rates of type 1a endoleak ranged from 2.3–22.6% [21,25,28,29]. In addition, one (1.6%) patient in the hTEVAR group experienced RTAD and died. RTAD originated from a partially clamped site of the ascending aorta and was not induced by the stent-graft. In some reports, an ascending aorta diameter of >40 mm and a stent-graft diameter of ≥42 mm are considered risk factors for RTAD [30,31,32]. Thus, we believe that using stent-grafts with a diameter of ≥42 mm is a risk factor for RTAD. However, patients in the bTEVAR group at high risk for median sternotomy reluctantly used 42–46 mm stent-grafts, none of whom had RTAD. The other endoleaks were observed in the bTEVAR group; however, we believe that these endoleaks could be prevented. Regarding bTEVAR, a shorter and stricter follow-up time seem necessary.

bTEVAR can be considered more minimally invasive because of its short operating time and postoperative hospital stay. Although bTEVAR may be effective for high-risk patients who are not candidates for median sternotomy due to its low risk of aorta-related death and aortic events, preventing cerebral infarction in these patients will be an important issue in the future.

### Limitations

This study has some biases as follows: (1) it was a retrospective single-center study with a relatively small sample size, (2) some patients had relatively short follow-up periods, and (3) the patients were carefully selected. Therefore, a prospective multicenter study with long-term follow-up is required to confirm our findings. Moreover, the findings of this study need to be validated through further clinical investigations.

## 5. Conclusions

We achieved satisfactory early and mid-term results of zone 0 landing bTEVAR and hTEVAR. bTEVAR might be superior to hTEVAR in that it is less invasive. bTEVAR may be considered an effective and more minimally invasive treatment for high-risk patients.

## Figures and Tables

**Table 1:** Patients’ characteristics and preoperative measurements.

	All *n* = 54	bTEVAR *n* = 25 (46.3%)	hTEVAR *n* = 29 (53.7%)	*p*-Value
**Patients’ characteristics**				
Age (years)	78 (73–82)	81 (76–84)	77 (69–80)	0.023
Age ≥ 80 years, *n* (%)	22 (40.7)	15 (60.0)	7 (24.1)	0.007
Female, *n* (%)	12 (22.2)	9 (36.0)	3 (10.3)	0.046
Emergency, *n* (%)	0	0	0	1.00
**Aortic pathologies**				
Degenerative aortic aneurysm, *n* (%)	49 (90.7)	22 (88.0)	27 (93.1)	0.653
Dissecting aortic aneurysm, *n* (%)	5 (9.3)	3 (12.0)	2 (6.9)	0.653
Dissection with patent false lumen, *n* (%)	0	0	0	1.00
**Medical history**				
Cerebrovascular disease, *n* (%)	12 (22.2)	7 (28.0)	5 (7.2)	0.513
Coronary artery disease, *n* (%)	11 (20.4)	7 (28.0)	4 (13.8)	0.310
CKD stage ≥ 4, *n* (%)	12 (22.2)	6 (24.0)	6 (20.7)	0.771
COPD, *n* (%)	14 (25.9)	12 (48.0)	2 (6.9)	0.001
EF (%)	66 (60–73)	65 (59–72)	68 (60–74)	0.419
Previous cardiovascular surgery, *n* (%)	13 (24.1)	10 (40.0)	3 (10.3)	0.023
Prior median sternotomy, *n* (%)	0	0	0	1.00
Logistic Euro SCORE (%)	32 (20–40)	38 (34–56)	21 (13–30)	<0.001

Data are represented as median (IQR: interquartile range). bTEVAR; branched TEVAR; hTEVAR; hybrid TEVAR; TEVAR: thoracic endovascular aortic repair; CKD: chronic kidney disease; COPD: chronic obstructive pulmonary disease; EF: ejection fraction.

**Table 2:** Preoperative measurements and stent-grafts.

	All *n* = 54	bTEVAR *n* = 25 (46.3%)	hTEVAR *n* = 29 (53.7%)	*p* Value
**Preoperative measurements**				
Maximum aneurysm diameter (mm)	58 (53–65)	57 (54–62)	60 (52–74)	0.335
Length of proximal LZ (mm)	33.6 ± 6.8	35.6 ± 1.3	31.9 ± 5.4	0.049
Diameter of proximal LZ (mm)	33.6 ± 3.0	34.9 ± 3.5	32.5 ± 2.0	0.003
Diameter of distal LZ (mm)	28.5 ±3.2	29.3 ± 3.4	27.8 ± 3.0	0.109
**Atheroma grade**				
Ascending aorta ≥2, *n* (%)	13 (24.1)	9 (36.0)	4 (13.8)	0.109
Aortic arch ≥3, *n* (%)	47 (87.0)	21 (84.0)	26 (89.7)	0.692
Descending aorta ≥3, *n* (%)	9 (16.7)	7 (28.0)	2 (6.9)	0.065
BCA ≥2, *n* (%)	14 (25.9)	6 (24.0)	8 (27.6)	0.764
Left CCA ≥2, *n* (%)	5 (9.3)	4 (16.0)	1 (3.5)	0.170
**Stent-grafts**				
Number of stent-graft, *n* (%)	1.5 ± 0.5	1.1 ± 0.3	1.8 ± 0.4	<0.001
Type of proximal stent-grafts				
Bolton Relay NBS, *n* (%)	25 (46.3)	25 (100)	0	
Bolton Relay Plus, *n* (%)	2 (3.7)	0	2 (6.9)	
Gore TAG, *n* (%)	10 (18.5)	0	10 (34.5)	
Gore CTAG, *n* (%)	16 (29.6)	0	16 (55.2)	
Cook Zenith TX2, *n* (%)	1 (1.9)	0	1 (3.4)	
Proximal stent-grafts				
Diameter (mm)	39.2 ± 3.8	41.9 ± 3.3	36.9 ± 2.4	<0.001
Oversizing rate (%)	16.9 ± 8.1	20.7 ± 8.1	13.7 ± 6.6	0.001
Distal stent-grafts				
Diameter (mm)	34.0 ± 3.9	34.4 ± 4.7	33.7 ± 3.1	0.531
Oversizing rate (%)	19.7 ± 8.6	17.6 ± 9.4	21.5 ± 7.4	0.097

Data are represented as mean ± standard deviation and median (IQR: interquartile range). bTEVAR; branched TEVAR; hTEVAR; hybrid TEVAR; TEVAR: thoracic endovascular aortic repair; LZ: landing zone; BCA: brachiocephalic artery; CCA: common carotid artery.

**Table 3:** Operative and in-hospital outcomes.

	All *n* = 54	bTEVAR *n* = 25 (46.3%)	hTEVAR *n* = 29 (53.7%)	*p*-Value
Procedure success (%)	100	100	100	1.00
Operative time (minutes)	255 (217–290)	220 (193–257)	279 (246–328)	<0.001
Postoperative hospital stay (days)	16 (12–25)	12 (9–22)	17 (14–26)	0.013
**In-hospital mortality**				
RTAD, *n* (%)	1 (1.9)	0	1 (3.4) *	1.00
**Aortic complication, *n* (%)**				
PND, *n* (%)	2 (3.7)	2 (8.0)	0	0.210
Spinal cord injury, *n* (%)	0	0	0	1.00
Abdominal embolic event, *n* (%)	0	0	0	1.00
New dialysis, *n* (%)	1 (1.9)	0	1 (3.4)	1.00
RTAD, *n* (%)	1 (1.9)	0	1 (3.4) *	1.00
Aneurysm enlargement, *n* (%)	0	0	0	1.00
Aneurysm rupture, *n* (%)	0	0	0	1.00
**Endoleaks, *n* (%)**				
Type 1a, *n* (%)	0	0	0	1.00
Type 1b, *n* (%)	0	0	0	1.00
Type 1c, *n* (%)	0	0	0	1.00
Type 2, *n* (%)	0	0	0	1.00
Type 3, *n* (%)	0	0	0	1.00

Data are represented as median (IQR: interquartile range). bTEVAR; branched TEVAR; hTEVAR; hybrid TEVAR; TEVAR: thoracic endovascular aortic repair; PND: permanent neurological dysfunction; RTAD: retrograde type A dissection, *: same patient.

**Table 4:** Late aortic events.

	All *n* = 54	bTEVAR *n* = 25 (46.3%)	hTEVAR *n* = 29 (53.7%)	*p*-Value
**Aortic complication, *n* (%)**				
PND, *n* (%)	0	0	0	1.00
RTAD, *n* (%)	0	0	0	1.00
Aneurysm enlargement, *n* (%)	0	0	0	1.00
Aneurysm rupture, *n* (%)	2 (3.7)	2 (8.0) *^,+^	0	0.210
Distal SINE, *n* (%)	0	0	0	1.00
Prosthetic infection, *n* (%)	2 (3.7)	0	2 (6.9)	0.493
Branched endograft occlusion, *n* (%)	0	0	0	1.00
Bypass graft occlusion, *n* (%)	0	0	0	1.00
**Endoleaks, *n* (%)**				
Type 1a, *n* (%)	0	0	0	1.00
Type 1b, *n* (%)	1 (1.9)	1 (4.0) *	0	0.463
Type 1c, *n* (%)	0	0	0	1.00
Type 2, *n* (%)	0	0	0	1.00
Type 3, *n* (%)	1 (1.9)	1 (4.0) ^+^	0	0.463

bTEVAR; branched TEVAR; hTEVAR; hybrid TEVAR; TEVAR: thoracic endovascular aortic repair; PND: permanent neurological dysfunction; RTAD: retrograde type A dissection; SINE: stent graft-induced new entry, *: same patient, ^+^: same patient.

## Data Availability

Data cannot be shared for ethical/privacy reasons. The data underlying this article cannot be shared publicly due to the privacy of individuals that participated in the study. On reasonable request, the data will be available from the corresponding author after approval from the Ethical Committee of the University of Osaka.
